# A Method Based on GA-CNN-LSTM for Daily Tourist Flow Prediction at Scenic Spots

**DOI:** 10.3390/e22030261

**Published:** 2020-02-25

**Authors:** Wenxing Lu, Haidong Rui, Changyong Liang, Li Jiang, Shuping Zhao, Keqing Li

**Affiliations:** 1 School of Management, Hefei University of Technology, Hefei 230009, China; 2 Ministry of Education Key Laboratory of Process Optimization and Intelligent Decision-Making, Hefei University of Technology, Hefei 230009, China

**Keywords:** prediction, daily tourist flow, genetic algorithm (GA), convolutional neural network (CNN), long-short-term memory network (LSTM), optimization method

## Abstract

Accurate tourist flow prediction is key to ensuring the normal operation of popular scenic spots. However, one single model cannot effectively grasp the characteristics of the data and make accurate predictions because of the strong nonlinear characteristics of daily tourist flow data. Accordingly, this study predicts daily tourist flow in Huangshan Scenic Spot in China. A prediction method (GA-CNN-LSTM) which combines convolutional neural network (CNN) and long-short-term memory network (LSTM) and optimized by genetic algorithm (GA) is established. First, network search data, meteorological data, and other data are constructed into continuous feature maps. Then, feature vectors are extracted by convolutional neural network (CNN). Finally, the feature vectors are input into long-short-term memory network (LSTM) in time series for prediction. Moreover, GA is used to scientifically select the number of neurons in the CNN-LSTM model. Data is preprocessed and normalized before prediction. The accuracy of GA-CNN-LSTM is evaluated using mean absolute percentage error (MAPE), mean absolute error (MAE), Pearson correlation coefficient and index of agreement (IA). For a fair comparison, GA-CNN-LSTM model is compared with CNN-LSTM, LSTM, CNN and the back propagation neural network (BP). The experimental results show that GA-CNN-LSTM model is approximately 8.22% higher than CNN-LSTM on the performance of MAPE.

## 1. Introduction

The deepening of the reform and opening and the rapid development of the national economy has simultaneously seen the improvement of the economic capacity and living standard of the Chinese people. An increasing number of Chinese people now focus on better quality of life and higher levels of spiritual pursuit. The recent years witness the government’s development of tourism. The development of the national tourism industry has been substantially promoted with various policies and activities. According to the 2018 report published by the National Tourist Bureau of China on the development of culture and tourism in 2018, the domestic tourism market maintained its steady growth; inbound tourism market grew slowly and steadily, whereas the outbound tourism market developed rapidly. Moreover, the total number of domestic tourists was 5.539 billion, inbound tourists was 141.2 million, outbound tourists was 149.72 million, and the total tourism revenue was 5.97 trillion yuan. In 2018, the total number of tourists was 6.024 billion of the 11,924 A-level scenic spots in China. The total tourism revenue of all A-level scenic spots had an increase of 7.8% over the previous year [1].

With the continuous evolution of information theory, system theory, and cybernetics, “information” has become an academic term and scientific concept and been applied to many fields of natural sciences and social sciences. Since the entropy value is a concept that indicates the degree of uncertainty or confusion of the system, the elimination of uncertainty means a reduction in the entropy value, information can also be called negative entropy [2]. Entropy has been widely used in the field of prediction. Mirna et al. [3] and Guan et al. [4] used the theory of entropy in predicting time series. Carles et al. [5] combined entropy with machine learning to predict macroeconomics. The current tourism information discipline system is developing rapidly, and tourism information as a development basis of tourism information science has also gained great development space. In her book “Tourism Information Science”, Sharda [6] described tourism information science from three aspects: travel recommendation system, community and user interface design. Tourism information refers to the general term for tourism-related materials and information, including tourist attractions, transportation, and weather. Because tourism information is complex and diverse, a tourism information flow will form in the tourism system. There is always the emergence and connection of information flows inside and outside the tourism system. It is necessary to use information theory to study the problem of information and information flow in the tourism system. Lu [7] defines the travel information flow as the exchange and dissemination of travel information related to travel activities and accompanying travel.

As early as the 1960s, scholars began to research the prediction of tourist flow, and many prediction models were proposed, such as classic time series models, econometric models, artificial intelligence models, and deep neural network models. So many research results have been achieved in theory and practice.

Classic time series models make predictions based on trends analysis about the past. They can highlight the role of time factors in prediction. However, classic time series models are sensitive to the accuracy of experimental data. Appearance of bad data will affect the experiment results. Meanwhile, classic time series models can only handle the linear data, not non-linear data. Classic time series models include autoregressive, moving average, autoregressive moving average (ARMA), autoregressive integrated moving average (ARIMA), and so on. Gustavsson and Nordström [8] predicted tourist flow in different areas by ARMA. Lim and McAleer used ARMA for monthly [9] and seasonal forecasts [10]. Econometric models are also extensively used in the prediction of tourist flow. The advantage of these models is that they can accurately draw causal correlations between all influencing factors and prediction. However, econometric models are generally considered from the perspective of economics and are directly applied for making assumptions. Consequently, they lack theoretical basis of empirical analysis. Econometric models include error correction model (ECM), vector autoregressive (VAR), autoregressive distributed lag model (ADLM), and almost ideal demand system (AIDS). Kim and Song [11] predicted tourism demand in Korea by ECM; they proved that the prediction of ECM is better than ARMIA and VAR for a limited account of time. S. I. Ao [12] used VAR and genetic algorithm (GA) and neural network (NN) to predict the tourism demand of Hong Kong. Lin et al. [13] predicted the number of outbound tourists of China by AIDS.

With the development of computer technology, artificial intelligence has been generally used in the prediction of tourist flow. Chen et al. [14] built a model on the basis of empirical mode decomposition and artificial neural networks to predict tourism demand. Law [15] extended the adaption of neural networks in tourist flow forecasting by integrating backpropagation neural network (BPNN) into non-linear and separable travel demand data. Pai et al. [16] built a hybrid model on the basis of support vector machine (SVR) to predict the number of tourists who travel between Hong Kong and Taiwan. In addition, some researchers study tourism prediction from new directions. Sun et al. [17] established a machine learning framework combined with online data (e.g., network search data), which proved that model combined with Baidu and Google search indexes can improve accuracy of prediction. Li et al. [18] used online data to predict the number of tourists in Beijing.

For daily tourist flow, many prediction models were proposed. Owing to the complex non-linear characteristics of the daily number of tourists on holidays and the obvious seasonal trend of holiday tourist flow, Chen et al. [19] proposed an approach which hybridizes SVR model with adaptive genetic algorithm (AGA) and the seasonal index adjustment, namely AGA-SSVR, to predict holiday daily tourist flow. In addition, holiday daily tourist flow data for Huangshan Mountain in China were employed as an example. The experimental results indicated that the AGA-SSVR model is an effective approach with more accuracy than the other alternative models including SVR model with adaptive genetic algorithm (AGA-SVR) and BPNN. Li et al. [20] proposed a BPNN model optimized by a fruit fly optimization algorithm (FOA) method that includes web search data to forecast daily tourist flow. The experimental results proved that compared with other prediction models, higher accuracy can be obtained when it comes to the peak season. Song et al. [21] proposed prediction method of optimized BPNN based on modified GA. They designed new chromosomes with multi-layer Step-structure, improve the encoding mode, fitness function and genetic operator, and introduce the self-adaptive crossover and mutation probability, which optimizes the network structure and initial network weights of BPNN synchronously. The experimental results proved that the nonlinear fitting and accuracy of the modified prediction methods are better than other prediction methods in prediction of daily tourist flow.

In predicting tourist flow, algorithms such as random forest (RF) and SVM are widely used, but each algorithm has its own disadvantages. RF makes the algorithm very slow due to the use of a large number of trees and cannot make real-time predictions. At the same time, RF often performs poorly when it encounters noisy data, and in tourism prediction, there are many and complex influencing factors, which will cause RF to be unsuitable. SVM also has the problem of slow operation speed. At the same time, it shows poor forecasting ability when the range of data changes is large. However, the tourist flow data usually fluctuates greatly with seasons, weather and other factors. The forecast performance did not meet expectations. Long-short-term memory network (LSTM) is very good at processing time-series data that it is used in predicting tourist flow. Li and Cao [22] used LSTM to predict tourist flow for the first time. They proved that LSTM could improve accuracy of prediction compared with ARIMA and BPNN, especially in the long term. Although, LSTM can fully reflect the long-term historical process about time-series data, it cannot mine the effective information and potential linkages between discontinuous data. To solve this urgent problem, new models based on LSTM were built. Khalid et al. [23] proposed a new Jaya-LSTM model to predict the future values of electricity demand and price. The hyperparameters of this algorithm are tuned using the Jaya optimization algorithm to improve the forecasting ability and increase the training mechanism of the model. Wei et al. [24] built CNN-LSTM model for short-time travel time prediction. CNN-LSTM model can play a role in other existing fields. Shen et al. [25] proposed a deep learning framework named Tensor-CNN-LSTM (TCL) to predict the travel time of a given path. Kim and Cho [26] proposed a CNN-LSTM model that can extract features to effectively predict the housing energy consumption. Chen et al. [27] predicted the intensity of typhoons by CNN-LSTM. Huang and Kuo [28] predicted particulate matter (PM_2.5_) CNN-LSTM. All those studies prove that CNN-LSTM model has excellent performance in prediction. On the basis of those studies, the current research scientifically selects the number of neurons of CNN-LSTM model by GA, which is scientific and efficient and makes CNN-LSTM model adaptive to predict tourist flow.

## 2. Related Methods

### 2.1. Genetic Algorithm (GA)

The genetic algorithm (GA) was proposed by Prof. John Holland [29]. He systematically explained the basic theories and methods of GA and proposed its most important schema theory. DeJong [30] successfully developed the GA into a robust, widely applicable, and efficient search technology. Subsequently, GA was widely used in many fields such as structural optimization, medical image processing, machine learning, and artificial intelligence.

**Figure 1:**
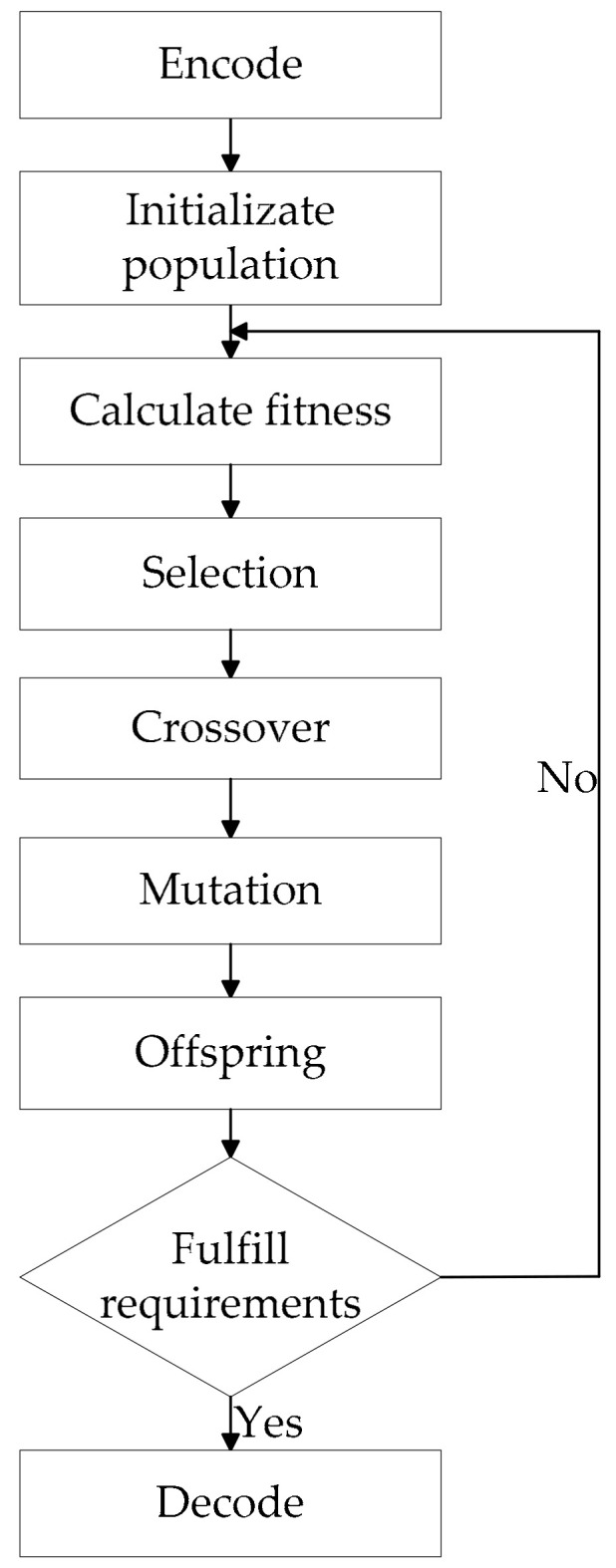
The GA flowchart.

Basic steps of standard GA include encode, initialization, selection, crossover, mutation, decode, and so on. First, coding is performed. After the generation of the first generation population, according to the principle of survival of the fittest, evolution from generation to generation produces better and better approximate solutions. In each generation, individuals are selected according to their fitness. And with the help of natural genetics genetic operators (genetic operators) to carry out crossover and mutation, to generate a representative new population. Figure 1 shows the GA flowchart.

### 2.2. Convolutional Neural Network (CNN)

Convolutional neural network was proposed by LeCun [31]. CNN is feedforward neural network. CNN employs local connection and weight share to extract feature of original data and builds dense and complete feature vector. This study uses CNN to extract data feature.

**Figure 2:**
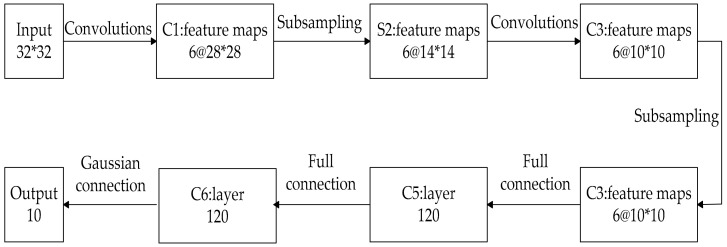
The structure chart of LeNet-5.

LeNet-5 is a typical CNN which includes input, convolutional layer, pooling layer, fully connected layer, and output. Figure 2 shows the structure chart of LeNet-5.

### 2.3. Long-Short-Term Memory Network (LSTM)

**Figure 3:**
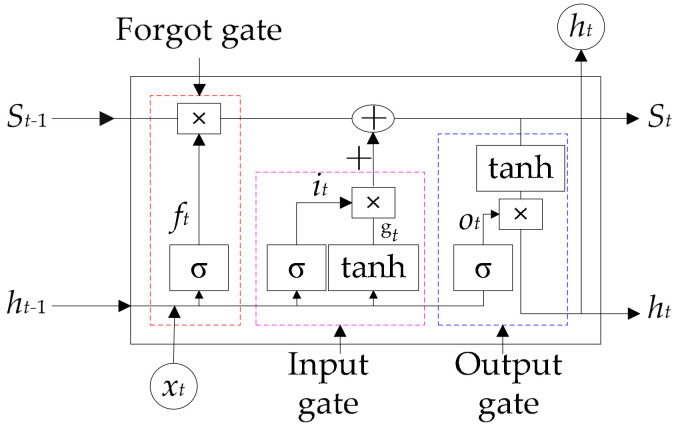
Basic unit of LSTM.

LSTM [32] is a modified Recurrent Neural Network (RNN). Compared with RNN, LSTM successfully solves gradient disappearance in training and long-term dependence in application by introducing memory cell and forgotten gate. Figure 3 shows its basic unit. 

The basic unit of LSTM includes forgotten gate, input gate, and output gate. Input (x_t_), state memory cell (S_t−1_), and mid-output (h_t−1_) jointly determine the forgotten part of state memory cell in forgotten gate. x_t_ determines the reserve vector in the state memory cell after sigmoid and tanh functions in input gate. Mid-output (h_t−1_) is determined by the updated (S_t_) and output (O_t_).

(1) $$f_{t}=\sigma \left(W_{f_{t}}+W_{f_{h}}h_{t-1}+b_{f}\right),$$

(2) $$i_{t}=\sigma \left(W_{i_{t}}+W_{i_{h}}h_{t-1}+b_{i}\right),$$

(3) $$g_{t}=\varphi \left(W_{g_{x}}+W_{g_{h}}h_{t-1}+b_{g}\right),$$

(4) $$O_{t}=\sigma \left(W_{O_{x}}x_{t}+W_{o_{h}}h_{t-1}+b_{O}\right),$$

(5) $$S_{t}=g_{t}⊙i_{t}+S_{t-1}⊙f_{t},$$

(6) $$h_{t}=\varphi \left(S_{t}\right)⊙O_{t},$$

where ${\;f}_{t}$, $i_{t}$, $g_{t}$, $O_{t}$, $h_{t}$ and $S_{t}$ are the states of forgotten gate, input gate, input nodes, output gate, mid-output, and state memory cell, respectively; $W_{f_{x}}$, $W_{f_{h}}$, $W_{i_{x}}$,${\;W}_{i_{h}}$, $W_{g_{x}}$, $W_{g_{h}}$, $W_{O_{x}}$ and $W_{O_{h}}$ are the matrix weight of the corresponding gates multiplied with input $x_{t}$ and mid-output $h_{t-1}$ respectively; $b_{f}$, $b_{i}$, $b_{g}$ and $b_{O}$ are the bias term of the corresponding gate; ⊙ represents point-wise multiplication; σ is sigmoid function; φ is tanh function.

## 3. Method Based on GA-CNN-LSTM

GA-CNN-LSTM hybrid model proposed in this study includes CNN for feature extraction, LSTM for prediction, and GA for optimization.

The main part of the method of prediction is CNN-LSTM. CNN module includes convolutional layer, pooling layer, and flatten. Convolutional layer (Conv2d) is set to m-layer. CNN can set the size of the convolution kernel to perform feature extraction on the data of different time periods. To maximize existing data, the size of the convolution kernel is set to n × n. At the same time, the number of convolution kernels of each convolutional layer is input into the genetic algorithm as the individual genes of the genetic algorithm. Pooling layer (Maxpooling2D) is set to m-layer, and the size of the pooling layer is set to n × n. The study adds batch normalization before the pooling layer to improve training efficiency. Then, the data are compressed by flatten to perform global feature extraction. Finally, the data are input to the LSTM module for prediction.

As learned through experiments, increasing the depth of the model by increasing the number of LSTM network elements helps improve the predictive capability of the model. Therefore, the LSTM module is set to the x-layer, and the number of neurons in each layer of the LSTM is input into the genetic algorithm as the individual genes of the genetic algorithm. LSTM uses Dropout to prevent overfitting. Finally, the vector of the specified format is output through Dense, that is, the daily tourist flow prediction value.

The GA-CNN-LSTM algorithm proposed in this article is an improvement on the CNN-LSTM algorithm. Regarding the CNN-LSTM algorithm, many papers have verified its effectiveness, especially in the field of prediction. The GA-CNN-LSTM algorithm uses the data extracted from CNN to input LSTM for prediction, and LSTM has unique advantages for prediction of time series data. In long-term time series prediction, LSTM will avoid the problem of gradient disappearance. However, for the selection of parameters in the CNN-LSTM model, most of the researches use manual selection methods such as grid search, which is time-consuming and laborious, and it is difficult to choose the most suitable parameters. Therefore, this article chooses GA, and uses the genetic and mutation process in GA algorithm for scientific selection of parameters, which will be more efficient from the perspective of time efficiency.

In this study, GA uses the process of selection, crossover, and mutation to choose scientifically the number of convolution kernels and neurons in each layer of the LSTM. By iterating and training the model until the preset number of iterations is reached or the requirements are met, the individual with the best fitness is obtained, and the genes of the individual are input as parameters into the CNN-LSTM to predict the tourist flow.

The individual fitness function is shown below:

(7) $$F=\frac{1}{E}\left(1+\alpha \left(1-\frac{n_{conv}}{n_{all}}\right)+\beta \left(1-\frac{n_{lstm}}{n_{all}}\right)\right),$$

where F is individual fitness; E is the error; this article uses MAPE as E; α is the influence of the convolution layer on the network performance; β is the influence of the LSTM on the network performance; in this article, α is 0.8, β is 0.2; n_conv_ is the number of convolution kernels; n_1stm_ is the number of neurons of LSTM; n_all_ is the total number of convolution kernels of CNN and neurons of LSTM.

In the GA-CNN-LSTM model, the individual fitness F and the MAPE have established an inverse proportional relationship, and the MAPE can be calculated to obtain the individual fitness, thereby evaluating the performance of the model and serving as the basis for the final parameters. From the perspective of time efficiency, this will take much less time than selecting all parameters of CNN-LSTM model with best performance by exhaustive method.

Based on the GA-CNN-LSTM model, this study proposes a complete modeling process to make predictions and evaluate the corresponding performance. The steps proceed as follows:

1. Preliminarily selecting factors related to the scenic spot, such as historical tourist flow data, meteorological data, tickets data, and so on. Correlation analysis is performed on historical tourist flow data, meteorological data, and tickets data. Moreover, high correlation is selected as input.
2. Selecting keywords of Baidu search index, which depends on what scenic spot the tourist is considering. Before traveling, tourists may search for information related to the scenic spot, such as weather, price, hotel, and so on.
3. Performing a correlation analysis between the keywords of Baidu search index which are obtained in Step 2 and tourist flow; selecting keywords with higher correlation as input.
4. Considering the lag of network search; setting a lag period and analyzing the correlation between Baidu index and tourist flow; choosing the lag period with the highest correlation.
5. Constructing a new data set that is input into the GA-CNN-LSTM model for daily tourist flow prediction.
6. Assessing accuracy of the GA-CNN-LSTM model; selecting relevant evaluation criteria for evaluation and comparing with related algorithms.

**Figure 4:**
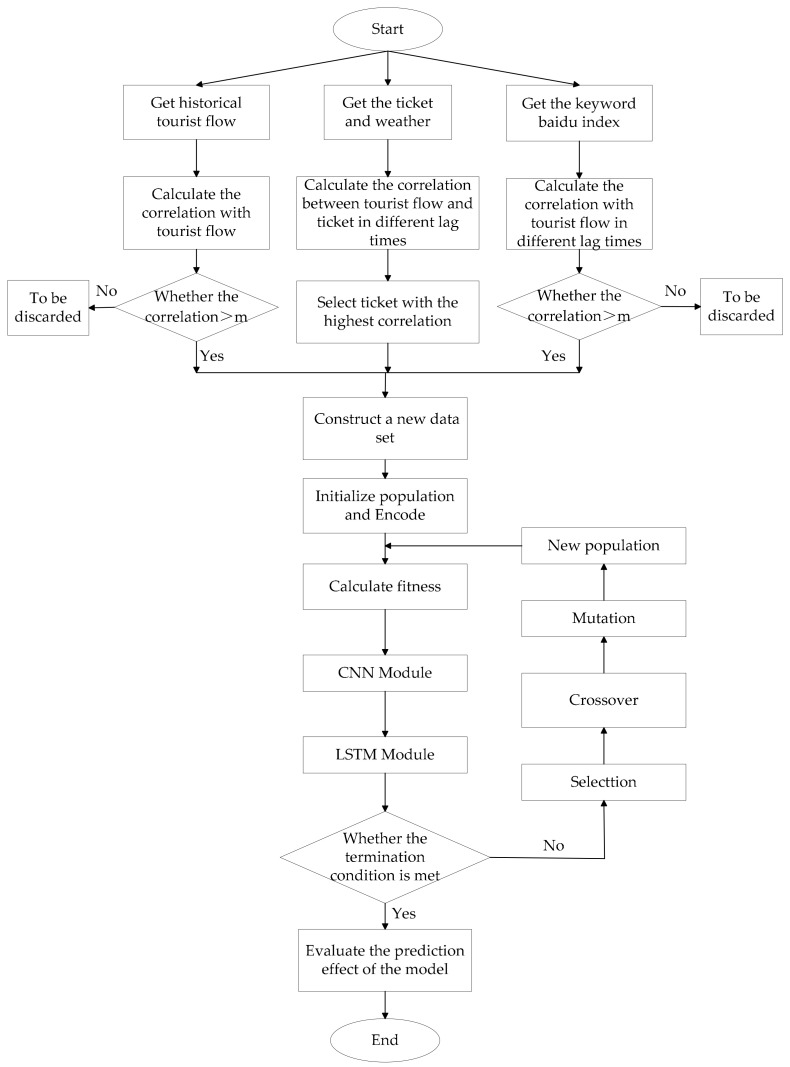
The research flow of this article.

Figure 4 shows the research flow of this article.

## 4. Empirical Study

### 4.1. Data Set Construction

This study takes China’s 5A-level scenic spot Huangshan as the research object and extracts features from related parameters to predict the change in tourist flow. It selects daily historical data from 2015 to 2018 as the original data. Each piece of data contains daily historical data of 16 related factors, which are divided into four categories, namely, tourist flow-related historical data, time factors, meteorological factors, and Baidu search index. All data come from a research project in cooperation with Huangshan Scenic Spot, web search index in Baidu search engine, and meteorological data from weather stations near Huangshan. Data correlation refers to the regularity between two or more variable values in a certain sense [33]; thus, correlation analysis is required for original data. 

Tourist flow-related historical data include tourist flow for 30 days ago, 365 days ago, same day last week, the day before yesterday, yesterday, and tickets data. Based on the correlation analysis between tourist flow-related historical data and target total number of tourists, this study selects data with correlation bigger than 0.4 that is tourist flow for 365 days ago, same day last week, the day before yesterday, and yesterday. Table 1 shows the correlation analysis results of tourist flow-related historical data and target total number of tourists with a confidence level of 0.01.

For tickets, considering the existence of lags in booking, the correlation analysis is performed on the tickets of different lag periods. Finally, the lag period with the highest correlation is determined as the number of tickets 1 day in advance, that is, the number of tickets yesterday. Table 2 presents the correlation analysis results of tickets and target total number of tourists with a confidence level of 0.01.

For tourist spots, the number of people on holidays and weekends is significantly more than weekdays, so the time factors are used as a key feature. In this article, the time factors are expressed by two characteristics. First, 1 to 7 is used to represent the day from Monday to Sunday, and 0 or 1 is used to represent whether the day is weekdays or holidays.

Huangshan is a mountain-type scenic spot. Meteorological factors will inevitably influence tourists’ decision. This study selects four meteorological factors, namely, weather, wind speed, average temperature, and average humidity. The weather uses 1–14 to represent 14 types of weather, namely, overcast, sunny, sunny to cloudy, shower, cloudy, light rain, moderate rain, heavy rain, rainstorm, light snow, moderate snow, heavy snow, blizzard, and sleet.

The selection and analysis of web search keywords are directly related to research that can accurately predict the tourist flow of scenic spots [34,35]. Based on searching the contents of scenic spots through the Internet before traveling, this paper selects relevant keyword benchmarks, such as, destination, tourist routes, maps, weather, food, tickets, etc. Based on the correlation analysis, this paper selects the five keywords with the highest correlation bigger than 0.4. Table 3 shows the results of the correlation analysis between keywords and total number of tourists with a confidence level of 0.01.

At the same time, considering the lag between information searched on the Internet and travel time. This paper analyzes the correlation between target tourist flow and keywords when the lag period is one day, two days, one week, 15 days and one month. Table 4 shows the correlation between total number of tourists and keywords under different lag period with a confidence level of 0.01.

According to the magnitude of the correlation under different lag periods, Baidu search index with lag period of 2 days is selected as an input.

To sum it up, this article uses tourist flow-related historical data, time factors, meteorological factors, and Baidu search index. Table 5 shows the specific characteristics.

The GA-CNN-LSTM hybrid model proposed in this study takes the time series feature map as the input. Tourist flow-related historical data, time factors, meteorological factors, and Baidu search index are independent time series. This study refers to the word vector method in natural language processing. The number of tourists at a time is represented by a series of related features into a vector; then, brand-new time series data are formed. The number of historical tourists at each moment is collectively represented by its related features. Then, the input time series data generated feature map is input into the CNN module by window sliding. To facilitate subsequent network calculations, the sliding window width is set to 16, the step size is set to 1, and the size of the unit feature map is 16 × 16. The input feature map is also arranged in time series. 

### 4.2. Data Preprocessing

To improve the prediction accuracy and stabilize the data before using GA-CNN-LSTM neural network for prediction, the original data sequences must be normalized to [0, 1]. This study uses the min-max normalization method, and the formula is shown below:

(8) $$x_{i}^{*}=\frac{x_{i}-x_{\min}}{x_{\max}-x_{i}},$$

where $x_{i}^{*}$ is the normalized data; x_i_ is the influencing factor of a certain tourist flow on day i, x_max_, x_min_ are the maximum and minimum values of the data of the corresponding sequence.

### 4.3. Data Set Partition

This article selects daily historical data of Huangshan Scenic Spot from 2015 to 2018 as the original data. Then, data from 2015 to 2017 are used as the training set; data from 2018 are used as the test set.

### 4.4. Experimental Environment

The experiments in this article are performed in the following hardware environment: CPU: Intel i5 9400f, Memory: 16GB, GPU: 1660ti. The software framework is a Tensorflow framework based on Keras deep learning tools, which is written by Python. Keras provides a simple and consistent programming interface that can help users quickly understand the neural network architecture and reduce the repetitive work in the code implementation process. Keras has the characteristics of modularity, and supports the free combination of model layers and layer-by-layer overlay.

### 4.5. Model Building

The constructed data set is divided into training and test sets, and the training set data are input into the model for training. The three-layer convolutional and pooling layers are selected according to the size of the unit feature map and the principle of the convolutional neural network. In the convolutional layer of the CNN module, the size of the unit feature map is 16 × 16, the size of the convolution kernel is set to 2, and the step size of the convolution kernel is set to 1. The pooling layer sets the same parameters as the convolutional layer. Then, weight of CNN is initialized. Meanwhile, CNN module also adds Dropout to reduce the probability of overfitting. The activation function is set to Selu (Scaled Exponential Linear Units) function [36]. Compared with traditional Relu (Rectified Linear Unit) function, the Selu function has better convergence performance and can effectively avoid the problem of gradient disappearance:

(9) $$Selu\left(x\right)=\lambda \left\{\begin{array}{c} \begin{array}{cc} x & x>0 \end{array}\\ \begin{array}{cc} \alpha e^{x}-\alpha & x\leq 0 \end{array} \end{array}\right.,$$

where λ=1.05; α = 1.67.

Some problems in deep neural network training are identified. For example, owing to the large number of layers in a deep neural network, changes occurring in the parameters of one layer will affect the output of all subsequent layers and result in frequent parameter modification and low training efficiency. In addition, before the data pass through the activation function, the output value of the nerve cell may also cause the failure of the latter to work if it remarkably exceeds the appropriate range of the activation function itself. Batch Normalization (BN) [37] is designed to solve this problem. In BN, momentum = 0.99. In this study, BN is added into CNN. The formula for BN is as follows:

(10) $$\mu_{B}=\frac{1}{m}\sum_{i=1}^{m}x_{i},$$

(11) $$\sigma_{B}^{2}=\frac{1}{m}\sum_{i=1}^{m}{\left(x_{i}-\mu_{B}\right)}^{2},$$

(12) $${\hat{x}}_{i}=\frac{x_{i}-\mu_{B}}{\sqrt{\sigma^{2}+\varepsilon }},$$

(13) $$y_{i}=\gamma {\hat{x}}_{i}+\beta \equiv BN_{\gamma ,\beta }\left(x_{i}\right),$$

where x_i_ is input value; y_i_ is the output value after BN; m is the size of the mini-batch, that is, a mini-batch with m inputs; $\mu_{B}$ is the average of all inputs in the same mini-batch; $\sigma_{B}^{2}$ is the variance of all inputs in the same mini-batch; next, obtaining the normalized ${\hat{x}}_{i}$ according to $\mu_{B}$, $\sigma_{B}^{2}$, ${\hat{x}}_{i}$, and formula (12), putting ${\hat{x}}_{i}$ into formula (13), and obtaining y_i_; γ and β are obtained through machine learning. Using BN can maximize the neurons in deep neural networks to improve training efficiency.

In the LSTM module, the weight is initialized using MSE as the loss function and Adam function as the optimizer, learning rate = 0.001, beta_1 = 0.9, beta_2 = 0.999. Here, our objective is to minimize the forecasting error of the model:

(14) $$MSE=\frac{1}{n}\sum_{i=1}^{n}{\left(y_{i}-{\hat{y}}_{i}\right)}^{2},$$

where n is the total number of samples in the test set; ${\hat{y}}_{i}$ is prediction result; $y_{i}$ is the number of tourists.

To keep the impartiality of performance evaluation, only the training data is used during the training, while the testing data is not used. Each time the training data are input to the GA-CNN-LSTM, a loss value is generated, according to which the optimizer uses a backpropagation method to adjust the parameters of GA-CNN-LSTM. The forecast result of GA-CNN-LSTM will be more and more accurate with the increase of training iterations. After the GA-CNN-LSTM training is finished, the testing data is input into the GA-CNN-LSTM, and the testing results and real results are compared to evaluate the performance of the GA-CNN-LSTM.

**Figure 5:**
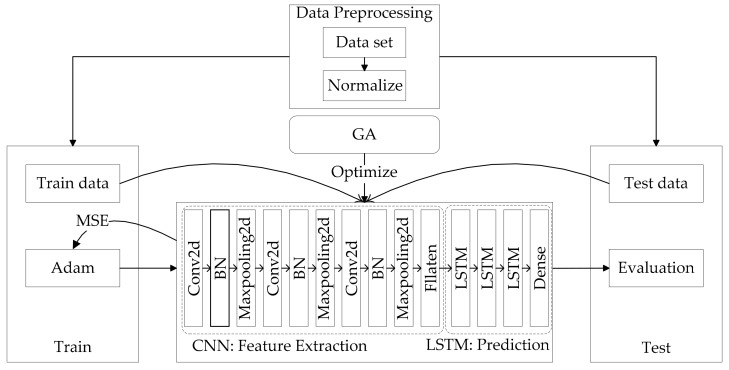
The specific training and prediction process of the model.

When there is not enough training data or when there is overtraining, overfitting may occur. However, there are many ways to avoid overfitting, such as regularization, data augmentation, dropout, dropconnect, or early stopping. The method used in this paper is dropout. CNN and LSTM module both add dropout to reduce the probability of overfitting. Figure 5 shows the specific training and prediction process of the model.

## 5. Result and Discussion

This study uses CNN-LSTM, CNN, LSTM, and BP as comparative experiments. The results of CNN-LSTM, CNN, LSTM, and BP will be compared with the experimental results of GA-CNN-LSTM. In addition, mean absolute percentage error (MAPE), root mean squared error (RMSE), Pearson correlation coefficient (r), Kling–Gupta efficiency (KGE) and index of agreement (IA) are used as the criteria for measuring the pros and cons of the model, with MAPE as the main evaluation criterion:

(15) $$MAPE=\frac{1}{n}\sum_{i=1}^{n}\left|\frac{y_{i}-{\hat{y}}_{i}}{y_{i}}\right|\times 100\%,$$

(16) $$r=\frac{n\sum_{i=1}^{n}y_{i}{\hat{y}}_{i}-\sum_{i=1}^{n}y_{i}\sum_{i=1}^{n}{\hat{y}}_{i}}{\sqrt{\sum_{i=1}^{n}y_{i}{}^{2}-{\left(\sum_{i=1}^{n}y_{i}\right)}^{2}}-\sqrt{\sum_{i=1}^{n}{\hat{y}}_{i}{}^{2}-{\left(\sum_{i=1}^{n}{\hat{y}}_{i}\right)}^{2}}},$$

(17) $$IA=1-\frac{\sum_{i=1}^{n}{\left(y_{i}-{\hat{y}}_{i}\right)}^{2}}{\sum_{i=1}^{n}{\left(\left|y_{i}-{\hat{y}}_{i}\right|+\left|\bar{y}-{\hat{y}}_{i}\right|\right)}^{2}},$$

(18) $$KGE=1-\sqrt{{\left(\alpha -1\right)}^{2}+{\left(\beta -1\right)}^{2}+{\left(r-1\right)}^{2}},\alpha =\frac{\sigma_{s}}{\sigma },\beta =\frac{\bar{\varepsilon }}{\bar{y}},$$

(19) $$RMSE=\sqrt{\frac{1}{n}\sum_{i=1}^{n}{\left(y_{i}-{\hat{y}}_{i}\right)}^{2}},$$

where n is the total number of samples in the test set; ${\hat{y}}_{i}$ is prediction result; $y_{i}$ is the number of tourists; $\bar{y}$ is the average of tourists. $\sigma_{s}$ and σ are *SD*s of prediction result and the number of tourists. $\bar{\varepsilon }$ is the average value of prediction result.

First, four CNN-LSTMs are manually selected with different numbers of neurons. Table 6 shows the number of neurons in four different CNN-LSTM models. 

Table 7 shows the comparison of MAPE GA-CNN-LSTM model and 4 different CNN-LSTM models under 5 experiments, respectively. For GA-CNN-LSTM model, MAPE_avg_= 20.77. For 4 different CNN-LSTMs, MAPE_avg_ = 23.08, 22.97, 22.89, 22.62, respectively. From the experimental results, the performance of GA-CNN-LSTM on MAPE can be concluded to be better than CNN-LSTM.

GA-CNN-LSTM, CNN-LSTM with the best performance, CNN, LSTM, and BP are selected as comparison experiments. Table 8, Table 9 and Table 10 show the comparison results of MAPE, MAE, r, and IA, respectively. From these tables, BP’s performance in tourist flow prediction can be seen as poor. CNN’s performance is better than BP, but a large error remains. Although a large error also exists, GA-CNN-LSTM and CNN-LSTM are more effective and accurate than LSTM. In addition, other specific details can be obtained from Table 8, Table 9 and Table 10. On the performance of MAPE, the average of the five experiments from small to large is GA-CNN-LSTM (20.77%), CNN-LSTM (22.63%), LSTM (25.12%), BP (28.18%), and CNN (29.81%). On the performance of r, the average of five experiments from small to large is GA-CNN-LSTM (0.913), CNN-LSTM (0.904), CNN (0.887), BP (0.887), and LSTM (0.844). On the performance of IA, the average of five experiments from small to large is GA-CNN-LSTM (0.919), CNN-LSTM (0.917), CNN (0.916), BP (0.904), and LSTM (0.889). From these data, on the performance of MAPE, GA-CNN-LSTM is generally better than CNN-LSTM and other algorithms, it is approximately 8.22% higher than CNN-LSTM, 16.68% higher than LSTM, 29.11% higher than CNN, and 26.59% higher than BP. On r, the performance of GA-CNN-LSTM is about 0.91% higher than CNN-LSTM, about 8.15% higher than LSTM, about 2.93% higher than CNN, and about 3.05% higher than BP. On IA, the performance of GA-CNN-LSTM is about 0.33% higher than CNN-LSTM, about 0.39% higher than LSTM, about 1.72% higher than CNN, and about 3.40% higher than BP. Although GA-CNN-LSTM performs better on r, and IA, none of them has made substantial improvements.

**Figure 6:**
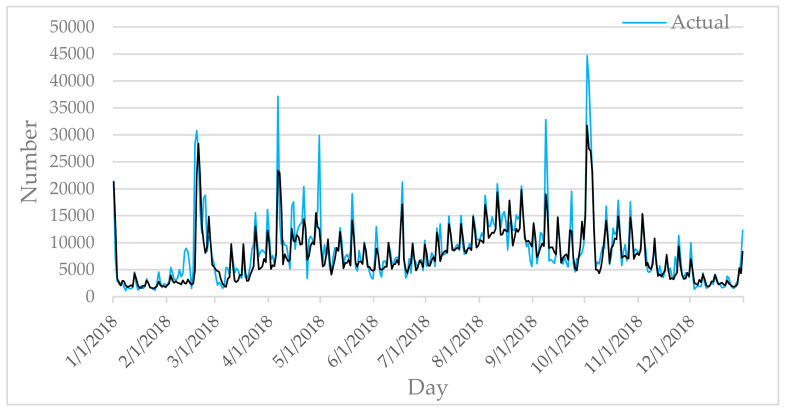
Comparison of GA-CNN-LSTM prediction results with Actual.

**Figure 7:**
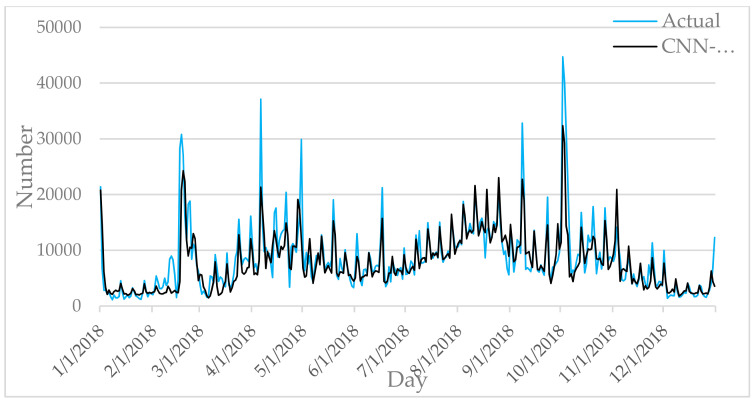
Comparison of CNN-LSTM prediction results with Actual.

**Figure 8:**
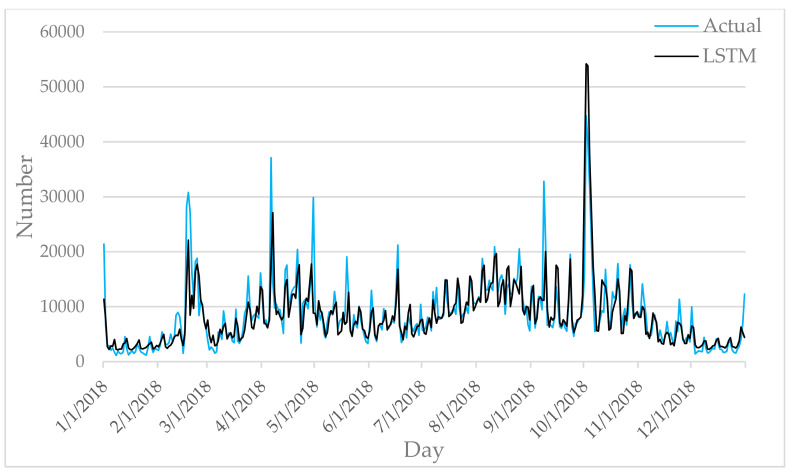
Comparison of LSTM prediction results with Actual.

**Figure 9:**
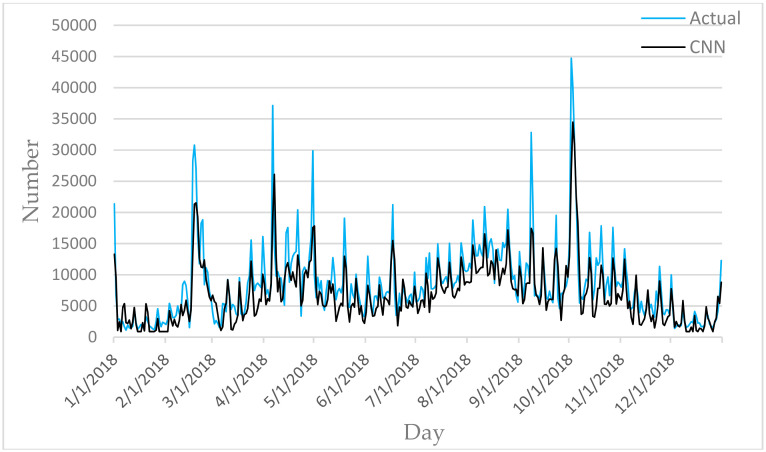
Comparison of CNN prediction results with Actual.

**Figure 10:**
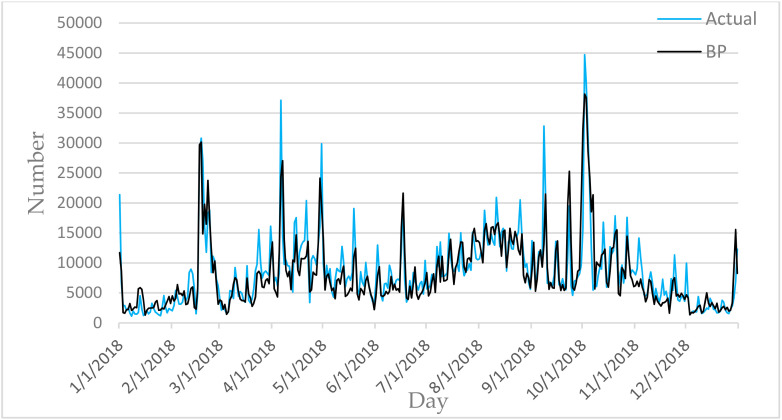
Comparison of BP prediction results with Actual.

Figure 6, Figure 7, Figure 8, Figure 9 and Figure 10 show the comparison between the prediction results of each algorithm in Test2 and the number of tourist flow. From these figures, the prediction results of BP and CNN algorithms can be summarized to fluctuate substantially than the number of tourist flow; a big gap exists between the changing trend predicted by BP and CNN algorithms and the changing trend of tourist flow; and the prediction results of the CNN algorithm are generally lower than the number of tourist flow. By contrast, GA-CNN-LSTM, CNN-LSTM, and LSTM show better prediction accuracy. GA-CNN-LSTM has an accurate grasp of the number of tourist flow throughout the year. However, the GA-CNN-LSTM, CNN-LSTM, LSTM, CNN, and BP algorithms all show inadequacy in predicting the number of tourist flow during peak time, and the prediction of the number of tourist flow in the peak time was generally low. In addition, the five algorithms have large errors in the predictions for January and February and thus require further research. 

On the performance of KGE in Test2, the results of the five algorithms from small to large is CNN (0.695), GA-CNN-LSTM (0.767), CNN-LSTM (0.772), LSTM (0.839), BP (0.872). KGE have an ideal value of 1. Therefore, from the perspective of KGE, GA-CNN-LSTM performs poorly. This is where improvement is needed. On the performance of RMSE in Test2, the results of the five algorithms from small to large is GA-CNN-LSTM (2983.33), CNN-LSTM (3185.16), CNN (3290.35), BP (3296.08), LSTM (3423.17). This proves that GA-CNN-LSTM is more accuracy.

Table 11 and Table 12 show the monthly and seasonal MAPE results of Test2. From Table 11, GA-CNN-LSTM’s prediction results can be seen as accurate in six months of the year, which shows the stability of GA-CNN-LSTM’s prediction. Evidently, GA-CNN-LSTM also has a series of problems. The performance is poor in February and March, and the prediction error is large. At the same time, during the peak season of tourism in Huangshan, the performances in July, August, and September are not as good as CNN-LSTM, BP, and LSTM. Nonetheless, the gap in error is not large. From Table 12, GA-CNN-LSTM can be seen to perform better than other algorithms in the first and fourth seasons. Owing to the geographical limitation of Huangshan Scenic Spot, the number of tourist flow in the first and fourth season often experiences considerable changes. However, GA-CNN-LSTM still shows better prediction accuracy, thereby demonstrating its efficiency for prediction in complex environments. 

On the predictions for the second and third seasons, although GA-CNN-LSTM is not the best, its performance still belongs to the acceptable range, and the gap among the best algorithms is small. For annual performance, GA-CNN-LSTM shows better stability than the others.

All these experimental results reflect the reliability and efficiency of GA-CNN-LSTM in tourist flow prediction. Although the prediction performance of both CNN and LSTM is good, GA-CNN-LSTM is better. It further proves that the method of extracting data features through CNN and predicting through LSTM is reliable.

## 6. Conclusions

Tourism has slowly become an important part of the local and national economy. How to manage scenic spot scientifically and efficiently is an urgent problem for the scenic spot management department. The prediction of tourist flow is the premise of management. Only under the premise of accurate prediction can the scenic spot management department make a reasonable allocation of scenic spot resources and ensure the sustainable development of the scenic spot. This study takes the famous Huangshan Scenic Spot as an example. It uses environmental, historical data, and Baidu search index to construct a new data set to express tourist flow and establishes a GA-CNN-LSTM-based prediction method. At the same time, considering the lag period between web search and travel, through the correlation analysis, the Baidu search index with the most relevant lag periods between keywords and the total number of tourist flow are selected. Compared with other algorithms, this method predicts daily tourist flow more accurately than the other intelligent algorithms in MAPE, r and IA. However, some limitations are identified in the experiment, which are worthy of further research. Examples include how to select influencing factors, pre-process data, and construct convolutional neural networks, and so on. Although the accuracy of GA-CNN-LSTM is higher than that of other algorithms during the peak time, the overall prediction accuracy of the peak time remains insufficient. In general, the GA-CNN-LSTM prediction method proposed in this study provides new ideas for daily tourist flow prediction. This method has a good prospect in tourism management research and application and can establish a healthy tourism industry and sustainable development.

## Figures and Tables

**Table 1:** Correlation Results.

Corresponding Rules	Yesterday	The Day before Yesterday	Same Day Last Week	30 Days Ago	365 Days Ago
Correlation	0.710	0.433	0.428	0.026	0.410

**Table 2:** Correlation Results.

Lag Period	1	2	3	4	5	5	7
Correlation	0.716	0.504	0.364	0.311	0.256	0.255	0.320

**Table 3:** Correlation Results.

Keywords	Correlation
Huangshan Scenic Spot	0.552
Huangshan Travel Guide	0.523
Huangshan Hong Village	0.636
Huangshan Travel Map	0.499
Huangshan Day Tour	0.585

**Table 4:** Correlation Results.

Lag Period	Correlation with the Different Lag Period				
	Huangshan Scenic Spot	Huangshan Travel Guide	Huangshan Hong Village	Huangshan Travel Map	Huangshan Day Tour
1	0.578	0.564	0.627	0.564	0.651
2	0.607	0.579	0.640	0.591	0.653
3	0.593	0.573	0.603	0.567	0.596
4	0.575	0.556	0.569	0.534	0.551
5	0.569	0.543	0.556	0.516	0.522
6	0.519	0.516	0.504	0.458	0.496
7	0.461	0.303	0.465	0.409	0.465
15	0.427	0.436	0.415	0.374	0.398
31	0.383	0.351	0.296	0.206	0.292

**Table 5:** The specific characteristics.

Impact Factors	Characteristics
Tourist flow related historical data:	The number of tourists yesterday; The number of tourists the day before yesterday; The number of tourists 365 days ago; The number of tourists Same day last week; The number of tickets.
Time factors:	Monday to Sunday; Holiday or Working day.
Meteorological factors:	Weather; Wind speed; Average temperature; Average humidity.
Baidu search index:	Huangshan Scenic Spot
	Huangshan Travel Guide
	Huangshan Hong Village
	Huangshan Travel Map
	Huangshan Day Tour

**Table 6:** CNN-LSTM with different neuron numbers.

CNN-LSTM (1)	CNN-LSTM (2)	CNN-LSTM (3)	CNN-LSTM (4)
64	32	64	32
128	64	128	64
256	128	256	128
16	32	32	16
32	64	64	32
64	128	128	64

**Table 7:** Comparison of GA-CNN-LSTM and CNN-LSTM (MAPE).

Test	MAPE (%)				
	GA-CNN -LSTM	CNN-LSTM (1)	CNN-LSTM (2)	CNN-LSTM (3)	CNN-LSTM (4)
1	20.73	22.96	23.71	22.52	22.90
2	20.50	22.93	22.71	22.77	22.29
3	20.86	23.71	22.89	23.80	22.56
4	20.79	22.62	23.10	22.96	22.64
5	20.96	23.18	22.43	22.41	22.74
Average	20.77	23.08	22.97	22.89	22.62

**Table 8:** The experimental results in terms of Mean Absolute Percentage Error (MAPE) (%).

Test	GA-CNN -LSTM	CNN-LSTM	LSTM	CNN	BP
1	20.73	22.90	24.92	29.81	28.36
2	20.50	22.29	23.96	29.81	28.56
3	20.86	22.56	26.64	29.80	28.33
4	20.79	22.64	24.54	29.81	29.02
5	20.96	22.74	24.56	29.81	27.18
Average	20.77	22.63	24.92	29.81	28.29

**Table 9:** The experimental results in terms of Pearson correlation coefficient (r).

Test	GA-CNN -LSTM	CNN-LSTM	LSTM	CNN	BP
1	0.911	0.908	0.847	0.887	0.875
2	0.912	0.900	0.842	0.887	0.887
3	0.912	0.908	0.847	0.887	0.889
4	0.916	0.901	0.846	0.887	0.874
5	0.912	0.905	0.837	0.885	0.903
Average	0.913	0.904	0.844	0.887	0.886

**Table 10:** The experimental results in terms of Index of Agreement (IA).

Test	GA-CNN -LSTM	CNN-LSTM	LSTM	CNN	BP
1	0.923	0.929	0.915	0.901	0.893
2	0.922	0.919	0.911	0.901	0.889
3	0.919	0.912	0.920	0.906	0.902
4	0.921	0.910	0.917	0.906	0.902
5	0.904	0.913	0.918	0.906	0.861
Average	0.919	0.917	0.916	0.904	0.889

**Table 11:** The experimental results in terms of MAPE per month (%).

Month	GA-CNN-LSTM	CNN-LSTM	LSTM	CNN	BP
1	24.9741	36.0873	46.7268	52.3842	62.7581
2	33.9763	36.3812	26.212	33.9085	33.4193
3	32.0348	33.182	32.3281	42.7591	23.8872
4	27.414	25.944	22.537	27.274	31.5567
5	15.8333	17.3235	16.4253	28.1739	22.1647
6	14.5825	15.5871	16.516	25.1302	22.4283
7	9.5686	8.4264	12.4824	18.8382	19.6289
8	15.7049	13.2258	14.6972	19.6488	12.7721
9	19.3528	16.9775	16.4402	20.7455	25.8335
10	15.0045	20.249	26.1285	29.4281	31.2337
11	14.8754	19.8404	18.0203	29.9235	24.6147
12	23.8101	25.2183	38.4586	29.3431	32.626

**Table 12:** The experimental results in terms of MAPE per season (%).

Season	GA-CNN-LSTM	CNN-LSTM	LSTM	CNN	BP
1	30.3284	35.2168	35.089	43.0172	40.0215
2	19.2766	19.6182	18.4928	26.8593	25.3832
3	14.8754	12.8766	14.5399	19.7442	19.4115
4	17.8741	21.7692	27.5358	29.5649	29.4915
